# Personal genomes to precision medicine

**DOI:** 10.1186/1755-8166-7-S1-I28

**Published:** 2014-01-21

**Authors:** Vinod Scaria

**Affiliations:** 1 GN Ramachandran Knowledge Center for Genome Informatics, CSIR-Institute of Genomics and Integrative Biology, Mathura Road, Delhi, India

## 

The decade after the draft Human genome was published has seen tremendous developments in the technologies that can sequence genomes of individual Humans in a fast and affordable and accurate way. Reading 3 billion odd bases which comprise of the Human genome is today possible in realistic timelines and costs. This improvement was primarily brought about by the significant advances in the throughput and technology to sequence DNA and computational methods and resources to assemble and interpret the data. In addition to the improvements in technology, the miniaturization of technology that enables sequencing of human genomes on bench top sequencers has been thought to be one of the game-changers which could potentially accelerate the widespread application of genome sequencing. The availability of the reference human genome sequence has also in the last one decade accelerated the discovery of human genetic variations and their associations with traits which has provided a basis of annotating human genomes for predictive/personalized medicine.

The availability of effective tools, resources and datasets has poised genome sequencing in a unique position which could see its regular use in clinical settings. The application of genomics in clinical settings would primarily help in accurate and evidence based care, which encompass a new field of medicine called Precision Medicine. Precision medicine encompasses use of accurate clinical information and evidence to appropriately manage a patient at an individual level or at a community level. One of the major challenges which preclude the widespread application of genome sequences in clinical settings is the lack of know-how and expertise in analyzing and interpreting genome data in clinical settings. This would require a new breed of clinicians who have good clinical acumen, and are equally well versed with genomics and computational tools and methodologies.

Towards accelerating the implementation of Precision medicine we have established a pipeline for human genome/exome sequencing and analysis in our laboratory and have developed a gamut of resources and tools for discovery, modeling and annotation of clinically actionable variants in genomes including Pharmacogenetic variations. In addition, we have implemented a pipeline for validation of functional variations in zebrafish, a popular vertebrate model system. Case studies and examples would be detailed during the lecture.

